# Emotional maturity and job satisfaction among healthcare employees the mediating and moderating roles of emotional intelligence

**DOI:** 10.1007/s44192-026-00410-x

**Published:** 2026-03-17

**Authors:** Debanjan Nag, Sangeetha Sukumaran, K. D. V. Prasad

**Affiliations:** 1 Symbiosis Institute of Business Management, Hyderabad, India; 2 Symbiosis International (Deemed University), Pune, India

**Keywords:** Emotional intelligence, Emotional stability, Emotional progress, Job satisfaction, Mediation

## Abstract

The proposed empirical research investigated the impact of emotional maturity on job satisfaction among healthcare employees. The study further examined the mediating and moderating roles of emotional intelligence in the nexus between emotional maturity and job satisfaction. A quantitative study was deployed, and nonprobability purposive sampling was used to select respondents working in the healthcare sector. The data were gathered via a structured questionnaire from healthcare professionals (doctors, nurses, paramedical staff and administrative staff) working in various hospitals and clinical settings across Hyderabad Metro, India. Valid data from 500 respondents were subjected to SEM analysis to measure eight reflective constructs, including four sub-dimensions of emotional maturity, three sub-dimensions of job satisfaction, and a one-dimensional emotional intelligence scale. The findings reveal that emotional maturity significantly enhances job satisfaction and that emotional stability, emotional progression, and social adjustment are good predictors of job satisfaction. Emotional intelligence is positive and significant, impacts job satisfaction and partially mediates the nexus among emotional maturity and employees’ satisfaction with leadership, teamwork, and rewards. Furthermore, emotional intelligence strengthens the positive relationship between emotional maturity and job satisfaction. The study underscores the importance of strengthening emotional competencies in healthcare settings, suggesting that targeted emotional intelligence and maturity-building interventions can improve satisfaction, reduce turnover, and enhance patient care quality. This research contributes to the emotional behavior literature by integrating maturity and intelligence within a higher-order SEM framework in the Indian healthcare context.

**Supplementary Information:**

The online version contains supplementary material available at 10.1007/s44192-026-00410-x.

##  Introduction

Cognitive and emotional functioning is a pivotal predictor employees psychological well-being and employee performance in high-volume and demanding healthcare service sector.

The quick decision making, continuous patient interaction, empathy with patients are characteristics of health care assignments, where doctors, nurses paramedical staff face such type of environment. Prolonged stress and demanding work environment is another characteristic of healthcare sector. Thus, there is a need for enhanced emotional competence beyond technical know-how. Healthcare workers in the United States, Asia and Europe reported increasing emotional exhaustion and burnout levels ranging from 25 to 50% across the regions in which they work (WHO, [47]). However, this phenomenon is not limited to these three regions, and similar patterns were observed in the healthcare staff of the United Kingdom, Australia and Southeast Asian countries, resulting in turnover due to emotional exhaustion. Medical errors and decreased patient satisfaction have also been reported in these countries. Emotional exhaustion in healthcare workers is a global concern, and there is an urgent need to understand emotional maturity and emotional intelligence issues that help healthcare staff regulate the effects of work demands, stress coping, and sustained professional satisfaction.

Although emotional maturity is an important factor, it is underexplored in existing organizational psychology in the context of healthcare research. However, emotional intelligence has been researched substantially across the globe, and emotional maturity, the ability to manage emotions and life stressors while exhibiting stability, self-control resilience and interpersonal adjustment in demanding situations were investigated in early psychology research; however, these studies were conceptually fragmented across regions. The cross-national research reveals inconsistent use of emotional maturity and weak amalgamation with modern emotional proficiency; however, it is limited to empirical research and testing of its outcomes at work. In addition, the association between emotional maturity and job satisfaction is frequently emphasized narratively in existing studies, with a weak theoretical understanding of the frameworks through which emotionally mature individuals perceive satisfaction in demanding environments. Global research has isolated emotional maturity as a personality trait rather than positioning it within broader affective theories such as affective events theory or broaden-and-build theory.

Additionally, global writings rarely investigate how emotional intelligence, a well-established predictor of psychological well-being and employee performance, can act as a mediator or moderator through which emotional maturity structures job satisfaction. While some cross-country studies underscore the positive effects of emotional intelligence on the regulation of stress and associated outcomes, the dynamic interplay among emotional maturity, emotional intelligence and job satisfaction remains largely underresearched. Most global researchers have investigated emotional intelligence and job satisfaction independently, not taking into account the hierarchical structure of emotional maturity. Therefore, this study provides limited evidence from emotionally intensive professions in the healthcare sector.

In the Indian context, such global gaps are magnified because of structural challenges such as high patient loads, limited healthcare staffing, and emotionally charged interactions. However, prevailing studies in the context of India have focused primarily on emotional intelligence alone and lack integrated models that examine both emotional maturity and emotional intelligence together. There is a need to develop and test a an inclusive framework that dissects the role of how emotional maturity influences job satisfaction and how emotional maturity and job satisfaction behave in the presence of a mediator and moderator emotional intelligence [16].

An integrated framework was provided to bridge such cross-country gaps through this study. The outcome further supplements the available literature in this important area.

1. Emotional maturity (EM) and job satisfaction (JS) modeled as higher order constructs.
2. Investigate the direct influence of emotional maturity on job satisfaction using SEM analysis.
3. Model emotional intelligence (EI) as both a mediator and moderator.
4. In the context of healthcare sector in emerging-economies provide empirical evidence so a cross-cultural understanding of emotional forces at healthcare industry can be dissected.

### Review of literature

Theoretical underpinnings.

### Affective events theory (AET)

Affective events theory [44] theorizes that workplace incidents prompt emotional reactions and influence employees’ attitudes and job satisfaction. Recent extensions of AET emphasize that emotional traits, such as emotional maturity and emotional intelligence, structure the way employees construe and regulate these emotional incidents [23, 28]. Emotionally mature individuals have greater stability, impulse regulations, and resilience; thus, they respond constructively to negative affective incidents and maintain positive attitudes even under stressful and highly demanding environments. This concurs with modern research revealing that emotionally stable employees experience fewer negative affective fluctuations with perceived higher satisfaction, especially in the healthcare sector, where patient volume and service demands are high [12].

Furthermore, AET posits that individuals’ emotional competencies work as regulators of emotions in the workplace. EI, which defines how employees perceive, interpret, and convert emotional incidents into adaptive responses, is a critical factor in healthcare settings. Thus, under the AET framework, emotional maturity promotes emotional stability, whereas emotional intelligence acts as a framework that translates such stable emotional reactions into increased job satisfaction.

### Broaden-and-build theory (BBT)

Broaden-and-build theory [15] posits that positive emotions enhance individuals’ thought–action inventories and build persistent psychological resources. Modern research [32, 46] reports that EI assists in the generation of positive emotions via accurate emotion perception, empathy, and regulation, enhancing well-being and engagement.

Employees with greater emotional maturity can reduce their negative emotions, and with greater emotional intelligence, employees experience positive feelings, resulting in job satisfaction. Emotional maturity provides a foundational emotional base, whereas emotional intelligence enhances resources that improve interpersonal interactions and resilience.

### Theoretical mechanisms linking EM, EI, and JS

The AET and BBT theoretical frameworks explain the constructs’ interrelationships.

*Emotional maturity* →* Job satisfaction (AET)*

- Emotionally mature individuals regulate instincts, endure emotional balance, and remain composed in stressful environments, thus reducing negative affective reactions and increasing satisfaction (leadership, teamwork, and recognition).
- Higher job satisfaction lowers employee burnout, in turn increasing emotional stability (Almasri et al. [5]).

*Emotional maturity* →* Emotional intelligence* →* Job satisfaction (Mediation framework)* Emotional maturity augments key elements of emotional intelligence, such as self-awareness, empathy, and emotion regulation.

- EI transforms emotional maturity into adaptive coping, beneficial communication, and positive social interactions.
- EI therefore mediates the influence of emotional maturity on job satisfaction [6, 10].

*Emotional intelligence* ×* Emotional maturity*→* JS (Moderation framework)*

- EI strengthens the positive effect of emotional maturity by assisting employees in applying emotional regulation strategies more effectively.
- Employees with high EM and EI exhibit the greatest resilience, teamwork, and leadership satisfaction.
- EI moderates the nexus between personality characteristics and job attitudes [26, 36].

### Emotional intelligence (EI): contemporary perspectives

Modern research has linked EI characteristics to psychological well-being, work engagement, and job satisfaction across cultural settings [7, 35]. A robust association exists between EI and job satisfaction [17, 30, 45]. Employees high in EI perceive fewer negative emotions, exhibit more proactive coping strategies, and have higher satisfaction [37].

### Emotional maturity (EM): emerging discussions

Emotional maturity has reemerged in the recent organizational literature, particularly in the context of emotional regulation and resilience. Emotionally mature employees exhibit greater conflict tolerance, stronger workplace relationships, and lower emotional exhaustion [28, 42, 43]. However, there is an urgent need for rigorous empirical integration of EM with EI and job satisfaction in the global context in general, using higher-order structural models in particular.

### Job satisfaction (JS): updated evidence

Job satisfaction is shaped by affective, cognitive, and relational experiences at work. Recent studies underscore leadership support, interpersonal relationships, and emotional climates as strong predictors of job satisfaction across healthcare systems [14, 25]. Emotional competencies also play a critical role in predicting job satisfaction. Job satisfaction is strongly influenced by emotional regulation, work-related affect, and emotional climate—mechanisms directly related to EM and EI [3, 31].

### Research gap

On the basis of the expanded theoretical and empirical literature, there is a need to bridge several gaps:

- A unified model of emotional maturity, emotional intelligence and job satisfaction is needed, as there is limited integration of these three variables at present.
- There is a need to study emotional maturity and job satisfaction modeling as higher-order constructs to unravel structural relationships. The present studies lack higher-order models.
- The healthcare sector is an emotionally demanding sector, and there is insufficient empirical evidence where emotional competencies are critical.
- There is a need to conduct research in Southeast Asian contexts, as most of the studies are limited to Western and East Asian contexts.
- Limited studies on emotional intelligence as a mediator and moderator.
- Obsolete conceptualization of emotional maturity with contemporary theories.

Few studies have investigated the effects of emotional intelligence and emotional maturity on job satisfaction in industries with higher emotional labor, such as the healthcare sector.

Research questions

- Q1: “How do the lower-order constructs of the emotional maturity and emotional intelligence dimensions relate to the dimensions of job satisfaction?”
- Q2: “What is the relationship between the higher-order constructs of emotional maturity and job satisfaction?”
- Q3: “How do the higher-order constructs of emotional maturity and job satisfaction relate to emotional intelligence?”

## Hypotheses development

The nexus among emotional maturity, emotional intelligence and job satisfaction can be theorized in existing affective and emotional competency mechanisms. Empirical research has investigated the direct effect of each lower-order dimension of emotional maturity on the lower-order dimension of job satisfaction. The indirect effects were tested with emotional intelligence as a mediator and moderator. This empirical research also modeled the two constructs of emotional maturity and job satisfaction as higher-order constructs (emotional progression, emotional stability, personality integration and social adjustment, the subdimensions of emotional maturity; rewards and recognition, teamwork and leadership, the subdimensions of job satisfaction). These methodologies are in line with modern organizational behavior theories, which underscore the holistic, integrative functioning of emotional traits rather than the isolated subdimensions of the constructs.

### Emotional maturity and job satisfaction

AET posits that employees’ stable emotional characteristics influence how they perceive and react to workplace demanding experiences and shape overall job attitudes [44]. Emotionally mature individuals are more resilient to volatile and demanding environments that are prone to stress, regulate negative emotional arousal efficiently, and maintain self-control in demanding situations. Recent theoretical studies have shown that emotional stability and self-regulation are pivotal for sustained job satisfaction in demanding environments such as the healthcare industry [12, 28].

**H1** Emotional maturity positively and significantly influences job satisfaction.

### Emotional intelligence and job satisfaction

Employees with high emotional intelligence experience more positive affect and harmony at work according to trait emotional intelligence [35] and broaden-and-build theory [15]. These emotional responses nurture engagement and psychological well-being, thus enhancing job satisfaction [8, 9, 24]. Modern emotional intelligence theories also suggest that emotionally intelligent employees experience fewer negative events, such as threats, reduced emotional exhaustion and increased satisfaction [7, 17].

**H2** Emotional intelligence positively and significantly influences job satisfaction.

Emotionally mature individuals develop greater self-awareness and empathy—core dimensions of EI—in turn, enhance workplace relationships and fulfillment [38]. Hence, EI partially explains *how* emotional maturity leads to improved job satisfaction, which is consistent with cascading models of emotional competence [24].

### Emotional intelligence as a mediator

Modern emotional intelligence theories also suggest that emotionally intelligent employees experience fewer negative events, such as threats, reduced emotional exhaustion and increased satisfaction [7, 17].

In other words:

- The EM provides the emotional foundation (stability, control, adaptability).
- EI provides functional capability (identifying, processing, and using emotions effectively).

**H3** Emotional intelligence mediates the relationship between emotional maturity and job satisfaction.

### Emotional intelligence as a moderator

The moderating role of EI aligns with theories of emotional regulation and adaptive functioning, suggesting that EI strengthens the positive influence of emotional maturity on job satisfaction. Employees’ constructive emotional responses are possible with high EI in high-stress or conflict-ridden environments [29]. Employees with low EI may fail to apply emotional understanding effectively and may not benefit from emotional maturity. Thus, EI acts as a boundary condition that determines when and to what extent emotional maturity enhances job satisfaction. Emotional regulation theory [18] and interactionist models of emotional competence propose that individuals with higher EI are more capable of augmenting the positive effects of their emotional traits. This finding is in line with recent findings that EI strengthens the effects of personality traits on job outcomes [26].

**H4** Emotional intelligence moderates the relationship between emotional maturity and job satisfaction.

## Methodology

This research employed a descriptive research design to investigate the relationships among emotional maturity, emotional intelligence, and job satisfaction among employees in the healthcare sector in Hyderabad, India. A nonprobability purposive sampling technique was used to select respondents, specifically healthcare professionals, including doctors, nurses, and administrative staff working in various healthcare institutions across the city. A structured questionnaire will be used as the primary data collection tool. The questionnaire, consisting of 35 items across 8 reflective constructs, will be created and distributed via Google Forms. Responses that are incomplete or erroneous will be excluded. A total of 500 valid responses were considered for SEM analysis.

### Demographic characteristics

The sample includes 261 (52%) male and 239 (48%) female respondents. The age groups in terms of years of distribution are 41% 20–30, 43% 31–40, and 16% 41–50 years The are 37% graduates, 42% postgraduates and 21% others indicating diversity in sample.

### Theoretical background

**Fig. 1:**
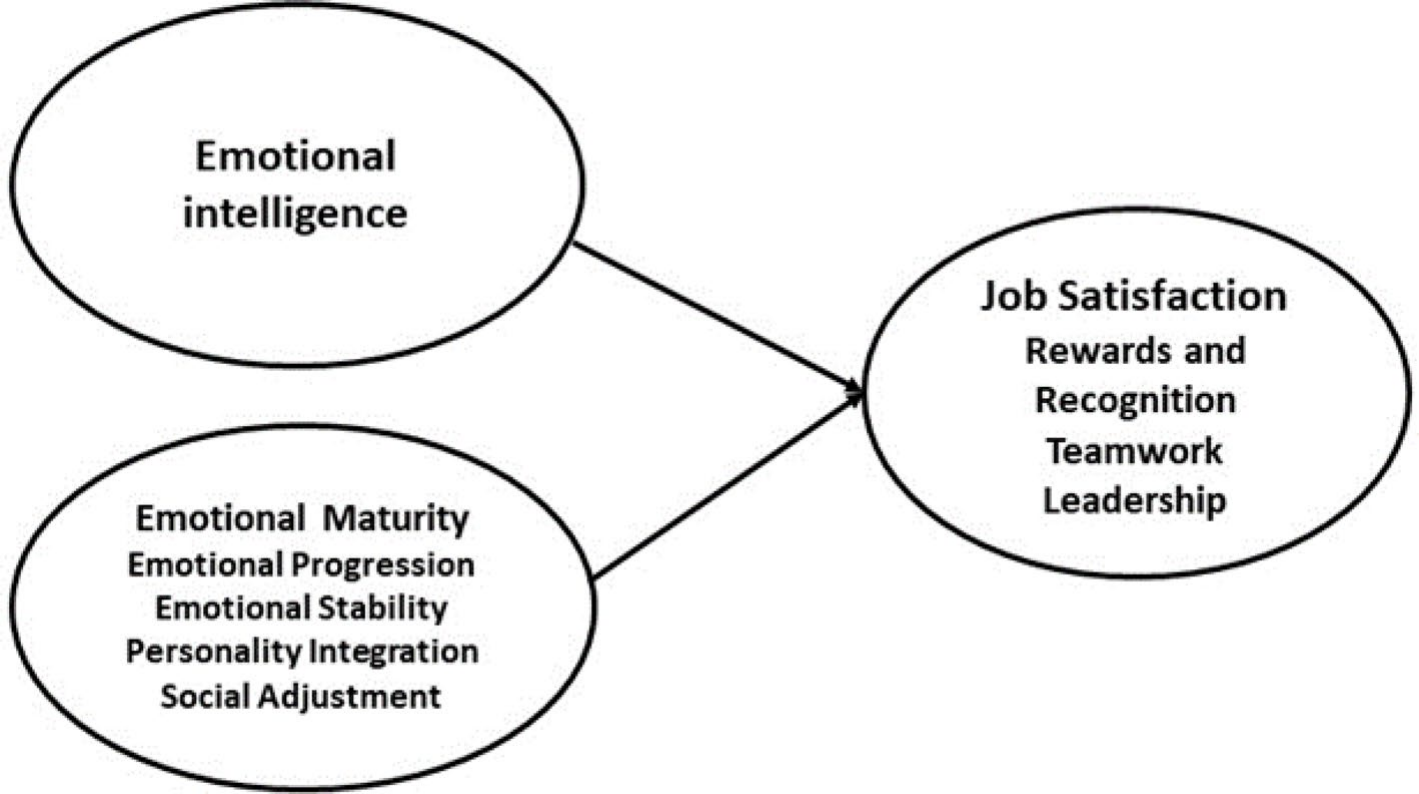
Theoretical framework (Authors’ creation)

**Fig. 2:**
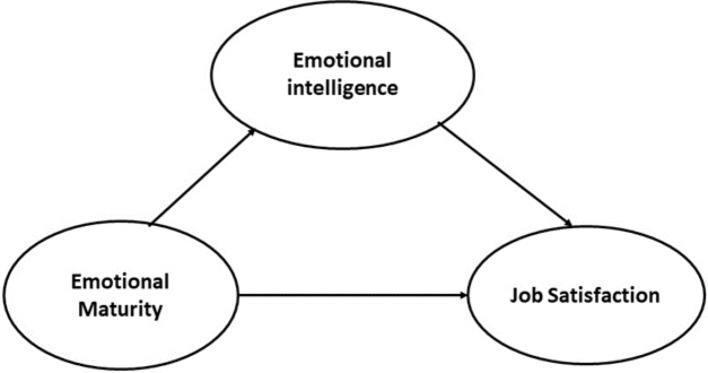
Mediation model (Authors creation)

The research study integrated emotional intelligence, emotional maturity and job satisfaction to develop a comprehensive framework. The emotional intelligence and emotional maturity are exogenous constructs, and job satisfaction is an endogenous construct. The model further tests the dual role emotional intelligence as mediator and moderato, and tested both the direct and indirect effects. The model was created following the methods of Schutte et al. [39], Singh and Bhargava [40] and Ahmad et al. [1]. The theoretical framework and mediation model are presented in Figs. 1 and 2.

### Measures

Emotional maturity will be measured via the scale developed by Singh and Bhargava [40], which includes four subdimensions: emotional stability (5 items), social adjustment (3 items), emotional progression (4 items), and personality integration (3 items), for a total of 15 items. Emotional intelligence will be assessed via a scale, and six items will be adapted from Schuttee, 1998). Job satisfaction will be measured via a questionnaire validated by Ahmad et al. [1], with three subdimensions—leadership (4 items), rewards and recognition (5 items), and teamwork (5 items)—for a total of 14 items.

###  Data collection

Data on 8 reflective constructs were collected via a structured questionnaire. The questionnaire, created as a Google Form, was distributed to employees in the healthcare sector. The respondents included doctors, paramedical staff, nurses, and other staff working in hospitals, laboratories, and clinics. A convenience sample was used; however, the data were collected from different age groups; different locations across Hyderabad city; and different small, medium and large corporate hospitals to avoid bias. A total of 570 completed questionnaires were received. However, 70 responses were not considered for the analysis because of incomplete responses and respondents’ misbehavior (selecting the same answer for all the statements). The demographic profile is presented in Table 1.

**Table 1:** Demographic characteristics of the study sample. *Source*: Primary data processed

Gender		Percent
Men	261	57
Women	239	48
*Age group*		
20–30	208	41
31–40	215	43
41–50	107	16

Education		
Graduate	185	37
Post-Graduate	214	42
Others	101	21

### Data analysis

Exploratory and confirmatory factor analyses were conducted on the collected data. Hypotheses were tested via SEM with IBM SPSS AMOS version 28. To investigate the relationships between the subdimensions of emotional maturity, emotional intelligence, and job satisfaction, both lower- and higher-order constructs are evaluated for reliability, discriminant validity, and model fit.

The reliability, validity, and model fit of both the higher- and lower-order constructs are assessed. Higher-order constructs, which are latent variables derived from multiple first-order latent variables, reflect broader, more comprehensive concepts that encompass several underlying dimensions.

The relationships among the constructs are analyzed via a two-stage approach. During the initial stage, measurement and structural models for every lower-order construct are evaluated. In the second stage, once the latent variable scores for the dependent lower-order constructs have been computed, the measurement and structural models of the higher-order constructs are analyzed.

##  Results

The factors loading for the study variables are presented in Table 2. The reliability, convergent and discriminant validity results are presented in Tables 3 and 4.

**Table 2:** Factor loadings of study variables

Item	Description	Factor loading
*Emotional Intelligence Chronbach’s α = 0.877, CR = 0.873, AVE = 0.586*		
EMOI1	“Emotions are one of the things that make my life worth living”	0.57
EMOI2	“I know when to speak about my personal problems to others”	0.66
EMOI3	“I like to share my emotions with others”	0.81
EMOI4	“I know why my emotions change”	0.89
EMOI5	“I easily recognize my emotions as I experience them”	0.85
*Emotional progression Chronbach’s α = 0.798, CR = 0.812, AVE = 0.520*		
EP1	“I do not put the blame on other for my lapses”	0.66
EP2	“On disagreement with others I do not start quarrelling with them”	0.77
EP3	“I am not self-centered”	0.72
EP4	“My behavior is more aggressive than the bahaviors of their friends”	0.73
*Emotional stability Chronbach’s α = 0.764, CR = 0.769, AVE = 0.555*		
ES1	“I have strong mental well-being”	0.61
ES2	“I am not stubborn”	0.69
ES3	“I do not get lost in imagination and daydreams”	0.88
ES4	“I do not stop at middle of any work before reaching the goal”	0.73
*Personality Integration Chronbach’s α = 0.880, CR = 0.881, AVE = 0.712*		
PI1	“I am not intolerant about the views of others”	0.83
PI2	“If I do not know about some work, I do not pose as if I know it”	0.88
PI3	“I do not easily my mental balance”	0.82
*Social adjustment Chronbach’s α = 0.887, CR = 0.840, AVE = 0.638*		
SOAD1	“I have a strained companionship with their friends and colleagues”	0.89
SOAD2	“I am not proud by nature”	0.81
SOAD3	“I do not hate others”	0.68
*Leadership Chronbach’s α = 0.871, CR = 0.848, AVE = 0.585*		
LEAD1	“My supervisor/senior manager visibly demonstrates a commitment to quality”	0.82
LEAD2	“It is clear to me what my supervisor expects of me regarding my job performance”	0.78
LEAD3	“My supervisor is able to address my questions or concerns”	0.72
LEAD4	“My supervisor has strong management skills”	0.73
*Rewards and recognition Chronbach’s α = 0.799, CR = 0.800, AVE = 0.512*		
RR1	“My base pay is fair for my responsibilities”	0.84
RR2	“The annual raise is reasonable”	0.86
RR3	“I am satisfied with the retirement plan”	0.56
RR4	“The process used to determine promotions/annual raises is just fair”	0.57
*Teamwork Chronbach’s α = 0.876, CR = 0.877, AVE = 0.643*		
TW1	“My coworkers are committed to doing quality work”	0.70
TW2	“It is easy to get along with my colleagues.”	0.84
TW3	“I feel part of a team in working toward shared goals”	0.84
TW4	“I experience a spirit of cooperation in this organization”	0.82

###  Main findings

Reliability and validity

IBM SPSS and AMOS 28 were used to assess reliability and validity following Hair et al. [21].

The internal consistency of the constructs was assessed by measuring Cronbach’s alpha (α) values and composite (CR) values, which exceeded the benchmark value of 0.70 for all the constructs [11].

Emotional Intelligence: α = 0.877, CR = 0.873

Emotional Progression: α = 0.798, CR = 0.812

Emotional Stability: α = 0.764, CR = 0.769

Personality Integration: α = 0.880, CR = 0.881

Social adjustment: α = 0.887, CR = 0.840

Leadership: α = 0.871, CR = 0.848

Rewards & Recognition: α = 0.799, CR = 0.800

Teamwork: α = 0.876, CR = 0.877

Convergent validity was assessed by measuring the average variance extracted, and the values for all the constructs exceeded the recommended value of 0.50.

Emotional Intelligence: AVE = 0.586

Emotional Progression: AVE = 0.520

Emotional Stability: AVE = 0.555

Personality Integration: AVE = 0.712

Social Adjustment: AVE = 0.638

Leadership: AVE = 0.585

Rewards & Recognition: AVE = 0.512

Teamwork: AVE = 0.643

The discriminant validity was assessed following the Fornell and Larcker [13] criterion and the heterotrait–monotrait ratio. The Fornell and Larcker criterion was met as the square of the AVE exceeded the interconstruct correlations, and discriminant validity was confirmed (Table 2). All the HTMT values were below the threshold of 0.85 [22] Table 4).

The exploratory factor analysis yielded eight valid components from the 35-item instrument after four items with factor loadings below 0.50 were excluded. The sampling adequacy (KMO = 0.867) and Bartlett’s test (*p* < 0.001) affirmed the data is suitable for SEM analysis. The eight-factors explained a cumulative variance 70.91%. The confirmatory factor analysis (CFA) exhibited excellent model fit (Chi square/df = 2.231, CFI = 0.951, TLI = 0.919, RMSEA = 0.054, SRMR = 0.036), validating the measurement model. The Cronbach’s α values ranged from 0.764 to 0.890 confirming the reliability and discriminant validity through average variance extracted, for all the construct AVE > 0.50; and heterotrait and monotrait analysis ratios (HTMT) are < 0.85.

**Fig. 3:**
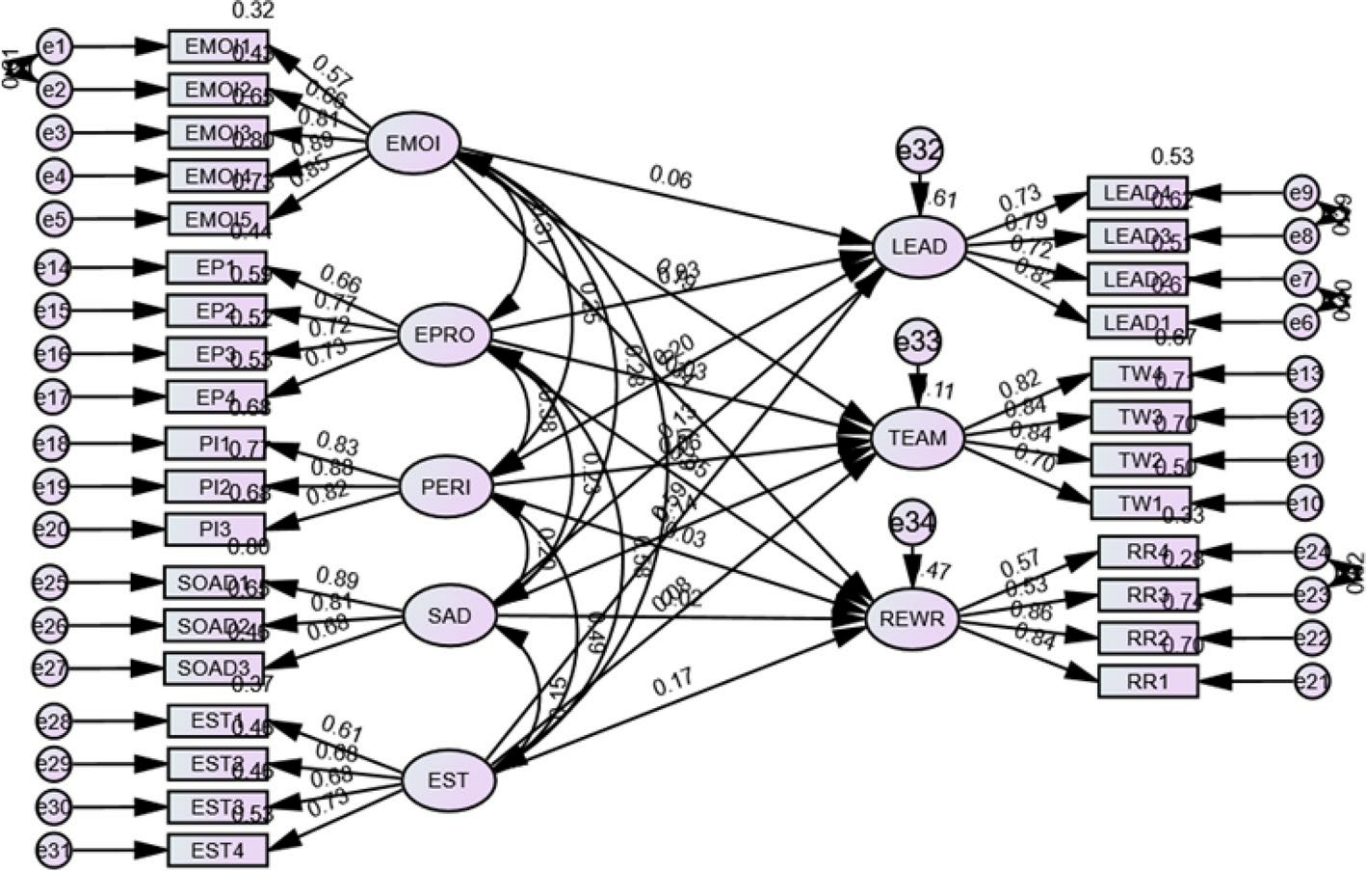
Structural model with relationship among the constructs

The SEM results revealed that emotional intelligence (EI) had a significant positive influence on leadership (β = 0.183, *p* < 0.001), teamwork (β = 0.204, *p* < 0.001), and rewards and recognition (β = 0.307, *p* < 0.001) the three sub-dimensions of job satisfaction. Among the emotional maturity (EM) sub-dimensions, emotional stability emerged as the strongest predictor of leadership satisfaction (β = 0.634, *p* < 0.01), followed by emotional progression (β = 0.336, *p* < 0.001) and personality integration (β = 0.146, *p* < 0.001). Social adjustment significantly influenced teamwork (β = 0.122, *p* < 0.05) and leadership (β = 0.098, *p* < 0.05). (Fig. 3).

### Theoretical interpretation of results

The SEM results reveal some important psychological and practical comprehensions of how emotional maturity and emotional intelligence shape job satisfaction among healthcare employees. We provide a discussion of the meaning, mechanisms and implications of each relationship.

*Emotional maturity* →* job satisfaction*

The SEM results reveal that emotional progression and emotional stability are strong predictors of job satisfaction in the context of leadership and rewards. Healthcare employees with emotional stability and progression maintain self-possession ability, recover emotionally after experiencing demanding and stressful situations and positively perceive the work environment.

*Psychological meaning*

The SEM results reveal that emotionally stable employees experience fewer negative emotional spirals when confronted with stressful incidents such as aggressive patients and medical emergencies, which is consistent with affective events theory. Whereas emotional progression assists employees in learning, adapting and increasing their emotional experience, these individuals can mentally reframe negative incidents, enabling their sense of meaning and satisfaction.

*Practical implications*

Healthcare centers with emotionally mature employees benefit from fewer emotional conflicts, enabling teamwork and better communication with peers.

Social adjustment → Job satisfaction

Social adjustment significantly impacts leadership and teamwork related to job satisfaction.

*Psychological meaning*

Healthcare employees with strong interpersonal adaptability construe peer feedback more practically and distinguish team dynamics more favorably. However, social adjustment decreases interpersonal strain and friction, enabling healthcare employees to focus on patient care despite team conflicts.

*Practical implications*

Training programs on conflict resolution and empathy drills can enhance social adaptability and substantially enhance team climate.

Emotional intelligence → job satisfaction

Emotional intelligence strongly predicts leadership, teamwork and rewards.

*Psychological meaning*

Healthcare employees with high emotional intelligence can easily predict peers’ expectations and forestall team requirements and can reframe reward inequality. Thus, emotionally intelligent employees perceive more positive emotions with high levels of engagement and satisfaction. This is consistent with broaden-and-build theory.

*Practical implications*

Emotional intelligence is a performance resource. Healthcare centers that invest in EI training expect more grains and better communication and satisfaction.

### Theoretical interpretation of the non-significant effects of personal integration

Personal integration (PI) did not significantly predict job satisfaction, and it offers meaningful psychological insights, as this is not a statistical artifact.

*The reasons*

Personal* integration* (PI) reflects internal coherence, not workplace functionality.

PI represents internal harmony, consistency among beliefs and actions, and self-congruence. Although desirable, personal integration may not directly influence how an employee perceives* external workplace experiences* such as leadership style, team climate, or reward fairness, whereas job satisfaction is an evaluation of the work environment—not the self.

*The healthcare* *sector is an emotionally turbulent environment where external demands override internal coherence.*

In high-stress and demanding jobs:

- Emotional stability matters more than internal consistency does.
- Social adjustment matters more than self-harmony does.
- Emotional regulation skills matter more than internal belief alignment does.

Thus, PI may be psychologically meaningful but not relevant to job satisfaction.

*Affective* *events theory predicts* *that job satisfaction is shaped by reactions to workplace events—not internal self-congruence.*

Personal integration may shape character or identity, but it does not necessarily change how one reacts to stressful healthcare events (e.g., patient conflicts, sudden emergencies, shift pressure).

*In the cascading model of emotional competence*,* personal integration is more distal.*

Cascading theory [24] proposes the following:

- Personality traits → emotion perception → understanding → regulation → performance.
- PI is a distal trait; its effects may be fully absorbed into EI and emotional stability, leaving no direct statistical effect.

### Comparison with previous research

These findings corroborate earlier studies linking emotional maturity with greater job satisfaction [20, 27]. Emotional stability is a strong predictor of positive leadership perceptions in demanding situations, which aligns with evidence that emotionally balanced individuals manage organizational stress more effectively. The positive impact of emotional intelligence on job satisfaction concurs with the outcome of Pathak et al. [34].

### Theoretical explanation

Theoretically, the results are in line with the affective events theory [44] and broaden-and-build theory [15]. Emotionally mature employees exhibit stability and adaptability, which nurtures positive affective experiences. Emotional intelligence, in turn, channels these emotional resources into effective interpersonal management and stress regulation, enhancing job satisfaction. The significant mediating effect of EI confirms that maturity alone is insufficient; employees must also possess the emotional skills to interpret and respond constructively to workplace emotions. The moderating influence of EI suggests that it amplifies the benefits of emotional maturity, particularly in emotionally intense healthcare environments.

**Table 3:** Discriminant validity (Fornell and Larcker criterion). *Source*: Primary data processed

	EMOI	LEAD	TEAM	EPRO	PERI	REWR	SAD	EST
EMOI	0.765							
LEAD	0.332***	**0.765**						
TEAM	0.276***	0.158**	**0.802**					
EPRO	0.309***	0.485***	0.191***	**0.721**				
PERI	0.250***	0.537***	0.188***	0.385***	**0.844**			
REWR	0.509***	0.487***	0.183***	0.570***	0.335***	**0.715**		
SAD	0.281***	0.281***	0.221***	0.228***	0.197***	0.222***	**0.799**	
EST	0.295***	0.733***	0.220***	0.577***	0.493***	0.472***	0.148**	**0.744**

EMOI, Emotional intelligence; LEAD, Leadership; TEAM, Teamwork, EPRO, Emotional progress; PERI, Personal integration; REWR, Rewards and recognition; SAD, Social adjustment; EST, Emotional stability ***Significant at <0.001 level

**Table 4:** Discriminant validity (heterotrait–monotrait analysis). *Source*: Primary data processed

	EMOI	LEAD	TEAM	EPRO	PERI	REWR	SAD	EST
EMOI								
LEAD	0.299							
TEAM	0.248	0.142						
EPRO	0.303	0.388	0.170					
PERI	0.211	0.462	0.184	0.322				
REWR	0.457	0.437	0.224	0.482	0.377			
SAD	0.273	0.243	0.209	0.205	0.165	0.241		
EST	0.261	0.574	0.191	0.456	0.419	0.410	0.128	

EMOI, Emotional intelligence; LEAD, Leadership; TEAM, Teamwork; EPRO, Emotional progress; PERI, Personal integration; REWR, Rewards and recognition; SAD, Social adjustment; EST, Emotional stability

**Table 5:** Testing of hypotheses. *Source*: Primary data processed

Relationship	ß	t- value	*p* value
Emotional intelligence →Leadership	0.183	3.101	.***
Emotional intelligence → Teamwork	0.294	3.384	***
Emotional intelligence → Rewards and recognition	0.307	6.512	***
Emotional progression → Leadership	0.336	4.36.	***
Emotional progression → Teamwork	0.043	0.401	0.688
Emotional progression → Rewards and recognition	0.321	5.604	***
Personality Integration → Leadership	0.146	3.868	***
Social adjustment → Teamwork	0.122	2.570	0.010
Social adjustment → Rewards and recognition	0.008	0.356	0.722
Emotional stability → Leadership	0.634	7.587	***
Emotional stability → Teamwork	0.109	1.119	0.263
Emotional stability → Rewards and recognition	0.125	2.563	0.010
Personality integration → Teamwork	0.053	1.014	0.310
Personality integration → Rewards and recognition	0.016	0.605	0.545
Social adjustment → Leadership	0.098	2.917	0.004

### Mediation analysis

To test the mediating effects of emotional intelligence on the nexus between emotional maturity and job satisfaction. Here, emotional maturity and job satisfaction were modeled as higher-order constructs. Before further analysis, researchers need to validate the higher-order constructs.

The validation of higher-order constructs subsequently occurred alongside that of lower-order constructs. We evaluated the reliability, convergent validity, and discriminant validity of the higher-order constructs. All three higher-order constructs have Cronbach’s alpha values greater than 0.70, which suggests reliability and consistency. Reliability, as well as convergent and discriminant validity, was evaluated (Fornel and Larcker, 1981 criterion), and HTMT analysis was performed. The constructs maintained their discriminant validity. Therefore, a mediation analysis was carried out.

###  Results mediation analysis

**Fig. 4:**
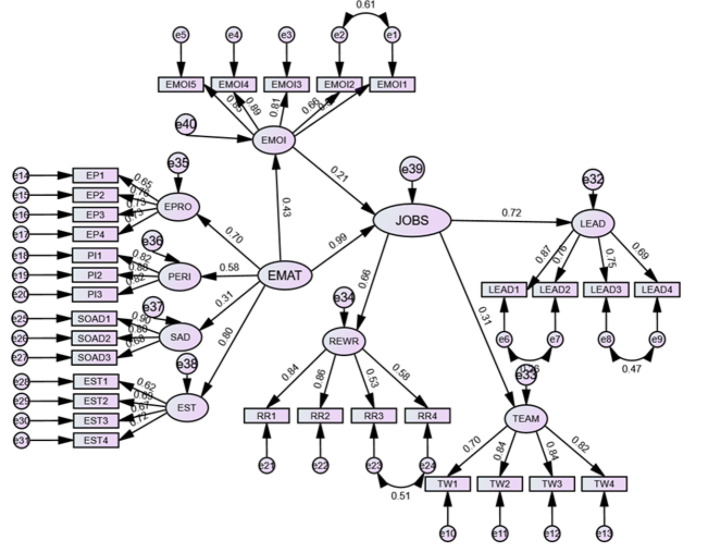
Mediation analysis

This study investigated the mediating effects of emotional intelligence on the relationship between emotional maturity and job satisfaction. The direct relationship between emotional maturity and job satisfaction is positive (ß = 0.436, t = 8.907). *p* < 0.001), and the indirect relationship is also positive and statistically significant (ß = 0.129, t = 3.101, *p* < 0.05; Table 6; Fig. 4), revealing that emotional intelligence partially mediates the relationship between emotional maturity and job satisfaction, partially supporting H_3_.

Emotional maturity facilitates effective emotional control in managing demands, whereas emotional intelligence transforms this control into constructive behaviors such as empathy, de-escalation, and improved communication, leading to greater satisfaction. This illustrates a dual process of stable emotions, emotional skills, and job satisfaction.

### Moderation analysis

A moderation analysis was carried out on the composite variables to further understand the nexus between emotional maturity and job satisfaction in the presence of a moderator. “The product term was created using independent and moderating variables, with mean centering applied to address high collinearity issues Aiken et al. [2]. A positive and significant moderating effect of emotional intelligence on the nexus between emotional maturity and job satisfaction was detected (ß = 0.195; t = 5.376, * p* < 0.001); thus, H4 was supported (Table 7).

High emotional intelligence is a characteristic of emotionally mature employees. Thus, these employees handle demanding situations that are emotionally intensive well. In turn, employees with low EI may exhibit emotional stability but find it difficult to apply their emotional characteristics appropriately. This aligns with emotional regulation theory [18], which posits that emotional traits require adequate regulatory strategies to handle positive workplace outcomes.

**Table 6:** Mediation analysis

Relationship	ß	t- value	*p* value
Direct effects			
Emotional maturity →Emotional Intelligence	0.43	6.052	***
Emotional intelligence →Job satisfaction	0.21	3.582	***
Emotional maturity → Job Satisfaction	0.99	8.907	***

Indirect pat	Unstandardized estimate	Lower	Upper	*P* Value
Indirect effects				
Emotional maturity → Emotional Intelligence → Job Satisfaction	0.129	0.053	0.260	0.008

**Table 7:** Results of the moderation analysis

Relationship	Beta	t value	*P*
Emotional maturity → Job satisfaction	0.587	7.875	*P* < 0.001
Emotional intelligence→Emotional maturity	0.287	5.175	*P* < 0.001
interEMCCxinterEICC → Job satisfaction Interaction between emotional intelligence and emotional intelligence on job satisfaction	0.195	3.376	0.007

**Fig. 5:**
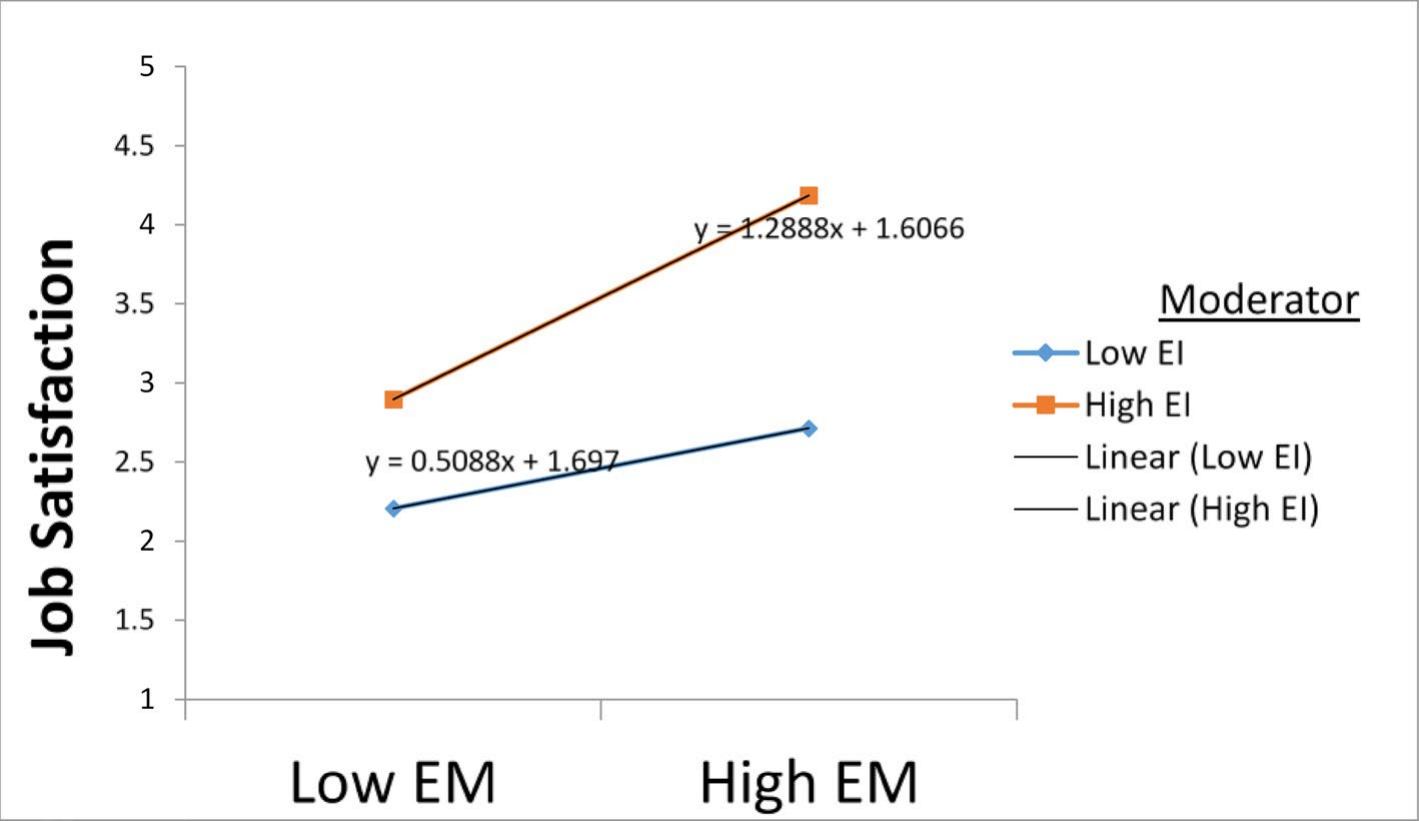
Emotional intelligence strengthens the positive relationship between emotional maturity and job satisfaction

The results of slope analysis reveal that high emotional intelligence strengthens the relationship between emotional maturity and job satisfaction. (Fig. 5, [33]).

## Discussion

This empirical study investigated the nexus between emotional maturity and job satisfaction among healthcare employees in Hyderabad and investigated the dual role of emotional intelligence as a mediator and moderator. The SEM results confirm that emotional maturity significantly influences job satisfaction in all three dimensions—leadership, rewards and recognition, and teamwork. Furthermore, emotional intelligence partially mediates this relationship, confirming its pivotal role in transforming emotional competence into positive workplace outcomes.

### Main findings and interpretation

The SEM results reveal that emotional maturity and its 4 subdimensions are good predictors of job satisfaction. Furthermore, emotional stability emerged as the strongest predictor of leadership satisfaction. Individuals who are self-regulated in stressful work environments perceive their leaders and organizational environment positively. These findings concur with those of Alessandri et al. [4], who reported that emotional stability safeguards job burnout and enhances job satisfaction. Emotional progression and social adjustment are vital in healthcare; thus, adaptability and interpersonal harmony are key psychological resources for effective teamwork and collaboration.

The EI partially mediated the nexus* between* emotional maturity and job satisfaction, suggesting that emotionally mature employees maintain self-control and easily adopt a new environment, resulting in increased job satisfaction. The outcome supports the findings of Guleryuz et al. [19] and Suleman et al. [41]. These researchers reported that EI is a key element linking emotional attributes to job satisfaction among healthcare professionals. Emotional intelligence enhances the positive impact of emotional maturity on job satisfaction. Therefore, emotionally intelligent employees can effectively utilize their maturity in emotionally charged environments.

### Theoretical implications

#### Refinement of emotional maturity theory

The outcome reveals that emotional maturity is not a uniformly predictive construct. The subdimensions of the constructs with outward behavioral relevance predict job satisfaction. However, the internally driven dimension (e.g., personal integration) is not a good predictor of job satisfaction, suggesting a reconceptualization of emotional maturity in organizational settings.

#### Integration of AET and broaden-and-build mechanisms

The results reveal that emotionally mature employees handle negative affective reactions positively (AET) and that emotionally intelligent employees generate more positive psychological resources (BBT), creating a dual-pathway emotional model of workplace satisfaction.

#### Dual-Role model of EI

EI serves as a psychological engine and performance amplifier, enhancing existing models and illustrating the intricate role of emotional capabilities in influencing workplace outcomes.

### Practical implications

1. Emotional Stability → Leadership Satisfaction (β = 0.634, *p* < 0.001)

The strongest predictor in the model was Emotional Stability influencing Leadership (β = 0.634). This indicates that emotionally stable healthcare employees are more likely to perceive supervisory support positively, particularly in high-pressure environments.

Practical Recommendation:

Hospitals should prioritize emotional stability development through:

- Stress inoculation training.
- Resilience-building workshops.
- Cognitive reappraisal training.
- Crisis-response simulations.

Given its large effect size, investing in emotional stability training is likely to yield the highest returns in leadership perception and supervisory climate.

2. Emotional Progression → Rewards & Leadership (β = 0.336; β = 0.321)

Emotional Progression significantly predicted leadership and rewards satisfaction. This suggests that employees who demonstrate adaptive growth and emotional learning perceive recognition systems more favorably.

Practical Recommendation:

Organizations should implement:

- Reflective practice sessions.
- Growth-mindset coaching.
- Emotional development mentoring programs.

These interventions may enhance satisfaction with reward systems by improving employees’ interpretive framing of workplace fairness.

3. Social adjustment → Teamwork (β = 0.122)

Social Adjustment significantly predicted teamwork satisfaction. This highlights the importance of interpersonal adaptability in healthcare teams.

Practical Recommendation:

Hospitals should conduct:

- Conflict resolution training.
- Interpersonal communication workshops.
- Collaborative simulation drills.

These initiatives are particularly important in multidisciplinary healthcare teams where coordination errors can affect patient outcomes.

4. Emotional intelligence → All three JS dimensions

Leadership (β = 0.183).

Teamwork (β = 0.294).

Rewards (β = 0.307).

Emotional Intelligence significantly influenced all three dimensions of job satisfaction and also acted as both mediator and moderator.

Practical Recommendation:

Healthcare institutions should:

Incorporate EI assessments into recruitment.

Offer structured EI development programs.

Train supervisors in emotion recognition and de-escalation techniques.

Because EI strengthens the EM → JS relationship (moderation β = 0.195), EI development amplifies the benefits of emotional maturity.

5. Personal integration (Non-significant findings)

Personal Integration did not significantly predict job satisfaction.

Practical Insight:

Organizations should focus more on outward-facing emotional competencies (stability, adaptability, social adjustment) rather than purely internal coherence traits when designing training programs.

## Conclusions

This study reveals several scientific contributions to emotional research and the healthcare human resource management literature. Empirical evidence has shown that emotional maturity is theoretically acknowledged but empirically underdeveloped—functioning as a second-order psychological reserve that shapes healthcare employees’ job satisfaction. By augmenting affective events theory with the cascading model of emotional competence, this study clarifies how stable emotional traits translate into work attitudes in emotionally demanding contexts.

Second, with the dual psychological mechanism of emotional intelligence as a mediator, emotional maturity influences behavior through functional processes, whereas as a moderator, it serves as a boundary condition that can enhance or diminish its benefits.

The study reveals that personal integration does not impact workplace satisfaction, questioning the belief that all facets of emotional maturity are equally vital for outcomes. In high-stress jobs, only externally oriented elements such as emotional stability, progression, and social adjustment are essential. This insight introduces a new theoretical approach for enhancing emotional maturity frameworks.

This study tests a higher-order SEM among Indian healthcare employees, extending global emotional competence research into an underrepresented emerging-economy context and thus enhancing cross-cultural validity and sector-specific theoretical relevance.

## Summary of limitations and future research

Despite its theoretical integration and empirical rigor, this study has several limitations that open meaningful avenues for future research.

*Cross-Sectional design and causality*

First, the study employed a cross-sectional design, which restricts causal inference. Although the 7structural equation modeling results support the hypothesized pathways among Emotional Maturity (EM), Emotional Intelligence (EI), and Job Satisfaction (JS), the temporal ordering of these constructs cannot be definitively established.

Longitudinal designs should be conducted to examine:

- Whether emotional maturity precedes the development of emotional intelligence over time.
- Whether EI training produces sustained increases in job satisfaction.
- How daily affective events dynamically influence satisfaction trajectories.

Experience sampling methodology (ESM) or diary-based designs could further unpack the micro-level emotional processes proposed by Affective Events Theory.

*Single-Source*,* self-reported data*

Second, all constructs were measured using self-reported questionnaires, raising potential concerns regarding common method bias and social desirability effects. Although reliability, discriminant validity (Fornell–Larcker), and HTMT criteria were satisfied, perceptual bias cannot be entirely ruled out.

Future studies should:

- Incorporate multisource data (e.g., supervisor-rated EI, peer-rated teamwork).
- Use objective indicators (turnover, absenteeism, patient satisfaction scores).
- Apply time-lagged data collection to reduce common method variance.

Such approaches would strengthen the robustness and external validity of the emotional competence model.

*Geographic and contextual boundaries*

Third, the study was conducted within the healthcare sector in Hyderabad, India. While this emerging-economy context enhances cross-cultural contribution, generalizability to other institutional environments may be limited.

Future research should:

- Conduct cross-country comparative studies.
- Test the model across public vs. private hospitals.
- Examine emotionally intensive sectors such as law enforcement, aviation, or hospitality.

Comparative studies would clarify whether emotional maturity functions similarly across cultural emotional norms and institutional climates.

*Construct scope and theoretical extensions*

Fourth, although emotional maturity was modeled as a higher-order construct, the non-significant effect of Personal Integration suggests that not all subdimensions operate equivalently in high-stress environments.

Future research may:

- Explore bifactor or network models of emotional maturity.
- Examine whether Personal Integration predicts distal outcomes such as ethical behavior, burnout, resilience, or meaning at work.
- Test nonlinear or threshold effects of emotional traits.

This would allow refinement of emotional maturity theory in organizational contexts.

*Mechanism expansion*

Fifth, the study focused on Emotional Intelligence as both mediator and moderator. While this dual-role model advances emotional competence theory, other psychological mechanisms may also explain how emotional maturity influences job attitudes.

Scholars may investigate:

- Psychological safety as a mediator.
- Burnout as a sequential mediator.
- Organizational climate as a moderator.
- Psychological capital as a complementary mechanism.

Such extensions would broaden the nomological network of emotional competence models.

*Practical and intervention-based research*

Finally, although the study suggests evidence-based training implications, the research did not experimentally test intervention effects.

Future research direction:

- Experimental or quasi-experimental designs could:
- Assess the effectiveness of EI training programs.
- Evaluate resilience-building interventions.
- Examine return-on-investment (ROI) of emotional development initiatives.

Intervention research would provide stronger translational impact for healthcare HR policy.

## Supplementary Information

Below is the link to the electronic supplementary material.

Supplementary Material 1.

## Data Availability

The data for this empirical research are available at the public platform Figshare at [https://figshare.com/s/2d3805f054a3929af67d] (https:/figshare.com/s/2d3805f054a3929af67d).
